# Streamlining the interface between electronics and neural systems for bidirectional electrochemical communication

**DOI:** 10.1039/d3sc00338h

**Published:** 2023-04-14

**Authors:** Wonkyung Cho, Sun-heui Yoon, Taek Dong Chung

**Affiliations:** a Department of Chemistry, Seoul National University Seoul 08826 Republic of Korea tdchung@snu.ac.kr; b Advanced Institutes of Convergence Technology Suwon-si 16229 Gyeonggi-do Republic of Korea

## Abstract

Seamless neural interfaces conjoining neurons and electrochemical devices hold great potential for highly efficient signal transmission across neural systems and the external world. Signal transmission through chemical sensing and stimulation *via* electrochemistry is remarkable because communication occurs through the same chemical language of neurons. Emerging strategies based on synaptic interfaces, iontronics-based neuromodulation, and improvements in selective neurosensing techniques have been explored to achieve seamless integration and efficient neuro-electronics communication. Synaptic interfaces can directly exchange signals to and from neurons, in a similar manner to that of chemical synapses. Hydrogel-based iontronic chemical delivery devices are operationally compatible with neural systems for improved neuromodulation. In this perspective, we explore developments to improve the interface between neurons and electrodes by targeting neurons or sub-neuronal regions including synapses. Furthermore, recent progress in electrochemical neurosensing and iontronics-based chemical delivery is examined.

## Abstract 2

Emerging strategies based on synaptic interfaces, iontronics-based neuromodulation, and improvements in selective neurosensing techniques have been explored to achieve seamless integration and efficient neuro-electronics communication.

## Graphical abstract

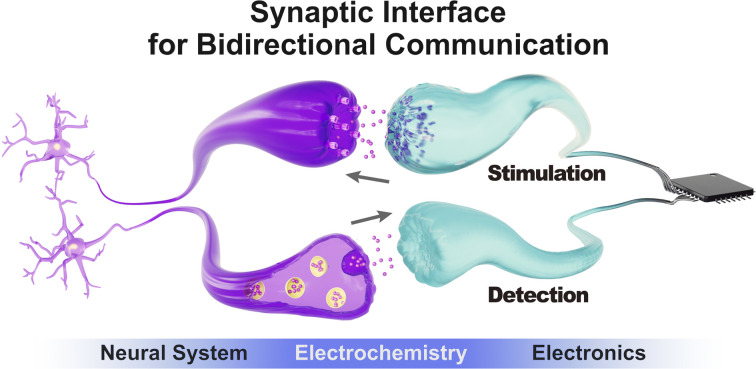

## 1. Introduction

Significant advancements in bidirectional information transfer between biological neural systems and the external world are being made possible by emerging developments in neural interface research. Such breakthroughs have implications for a wide range of research fields including neurochemistry, regenerative medicine, neuro-prosthetics, and wearable devices. A neural interface is a junction connecting two intrinsically different entities consisting of a lipid membrane-bounded neural system and a solid-state electronic circuit. Electrochemical techniques for the injection or uptake of electrical charge can drive the flow of signal carriers of neural signal transmission to integrate neural systems and electronics in a singular closed circuit. This type of system is referred to as the electrochemical neural interface, in which seamlessly integrated neurons and electrodes can mediate efficient electrochemical communication with neurons.

Our brains possess a myriad of synapses, which are sophisticated units for fast signal transmission and processing. The synapse is a subcellular region of neurons evolved for efficient interneuron relay of chemical information by diffusion of neurotransmitters from the presynaptic terminal to the postsynaptic membrane across a narrow cleft of about 20 nm.^1^ One promising direction for research in electrochemical neural interfaces is the development of methods for transmitting signals that utilize endogenous mechanisms in the brain, such as synaptic transmission. The subgroup of neural interfaces integrating synapses with external devices can be referred to as synaptic interfaces. Despite being in the early stages of development, synaptic interfaces show promise for robust and efficient bidirectional communication due to their similarities with biological synapses. This is one of many emerging trends in electrochemical neural interfaces that employ biohybrid strategies to enhance interfacing with biological systems.^2^

Two modalities, neurosensing and neuromodulation, are required for bidirectional communication in electrochemical neural interfaces. Signal transmission in a network of neurons occurs through rapid transfer of minute amounts of neurochemicals that results in changes in membrane potential. Electrochemical methods provide a suitable neurosensing platform due to their high sensitivity and high temporal resolution. However, challenges against long-term and *in vivo* electrochemical monitoring of the brain exist. A major hurdle is degradation of the neural interface due to foreign body response and electrode fouling. Another issue is signal selectivity due to the wide range of chemical compounds existing in the extracellular space of brains. Neuromodulation through the delivery of neurochemicals to neural systems is a rapidly growing field. Recently, chemical delivery devices based on iontronics have gained much traction due to their operational compatibility with neural systems in which both systems undertake signal processing by ions and molecules in an aqueous environment.

This perspective explores electrochemical neural interfaces in two aspects. The first focuses on recent progress made in the construction of intimate interfaces between neurons and abiotic substrates. Important considerations for neural probe design for use in long-term *in vivo* studies are also discussed. The latter section focuses on electrochemical monitoring and modulation of neural systems through the transfer of neurochemicals *via* iontronic devices.

## 2. Seamless integration of neurons and electrodes

For efficient communication with neurons, an ideal neural interface achieves seamless integration. Seamless integration refers to a tight and cohesive connection between artificial substrates and biological cells. In this perspective, we explore two aspects of seamless integration. First involves narrowing the physical distance between the neuronal membrane and electrode surface for improved exchange of chemical and electrical signals.^3^ Shorter the distance, minimal loss of signal carriers can be attained. Second, a stable connection must be secured for long-term durations in a complex cellular environment. The composition of cells at the neural interface can change over time due to neuronal migration and foreign body response. Neuronal migration is a common phenomenon during brain development^4^ and foreign body response is the occurrence of glial encapsulation and apoptosis of neurons near the neural probe. Together these processes can impede reliable communication with neurons as the electrode-neuron interface is degraded.

A major challenge against seamless integration is the inherent differences between abiotic electrodes and neurons in physicochemical compositions and mechanical properties. To bridge this disparity, research efforts attempt to engineer neural probes to conform to neurons. This section will focus on interfacing strategies for the formation of a tight neuron-electrode junction and targeting subcellular areas of neurons. Furthermore, other important considerations for the design of neural probes will be explored for improved neural interfacing.

### 2.1 Integration with a non-specific neuronal membrane

#### 2.1.1 Effect of surface topography of substrates

Research in contact guidance has shown that neurons react to surface topography on the nano- to micro-scale, which is similar in size to cellular sensing organelles such as filopodia and axonal growth cones.^5–7^ Furthermore, advances in lithography techniques with nano-resolution control paved the way for rigorous studies on the effect of surface topography on cellular behavior with significant implications for neural interfacing.

The influence of substrate topography on cells has been analyzed in relation to the membrane curvature. When cells are placed on nanostructures, they exhibit a local membrane curvature in a feature size-dependent manner.^8^ Computational simulations have been conducted to investigate the impact of various surface parameters on cellular adhesion to nanostructures. The structure radius, distribution density of nanostructures, and roughness ratio are some parameters discussed.^9,10^ The control of electrode surface topography can autonomously drive cellular processes, including cytoskeleton rearrangement for cell adhesion, growth and development.

**Fig. 1:**
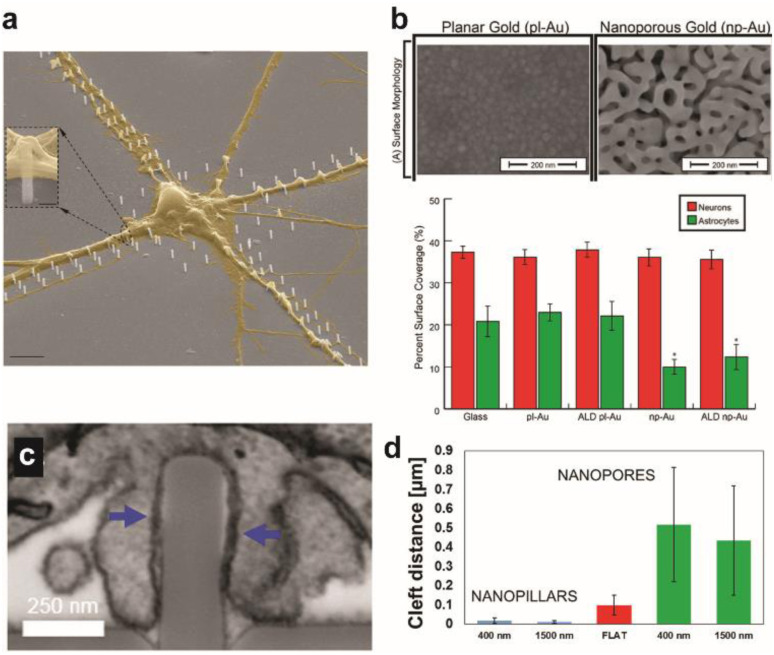
Effect of surface topographical features on neuronal cultures. (a) Colored SEM images of the guided culture of neurons by the geometric patterning of a nanopillar coated with poly-dl-ornithine (PDLO). Scale bar: 4 μm. Adapted from ref. 11 with permission from the American Chemical Society, copyright 2018. (b) SEM images of planar gold and nanoporous gold surfaces. Scale bar: 200 nm. Bar graph showing the surface coverage of neurons and astrocytes on various substrates. Adapted from ref. 19 with permission from the American Chemical Society, copyright 2015. (c) Surface topography affects the cleft distance between the cell membrane and underlying surface. FIB-SEM cross-sectional image showing the wrapping of the plasma membrane around nanopillars. Scale bar: 250 nm. (d) Cleft distance measurements show a drastically smaller cleft distance with nanopillars than nanopores or flat surfaces. Adapted from ref. 14 with permission from the American Chemical Society, copyright 2017.

Amin *et al.* reported guided growth of neurites along patterned routes of vertical nanopillars.^11^ Functionalization with poly-dl-ornithine on nanopillars enhanced synapse maturation compared to flat surfaces as a demonstration of the combinatorial effect of nanotopographical and biochemical cues on synaptic development (Fig. 1a). Gautam *et al.* observed alignment of neurites from different neurons at vertical nanowires thereby increasing the probability of synaptic connections in the neuron network, which exhibited synchronized calcium activity.^12^ The polarity of neurons, such as the cellular distinction between soma and axon, as well as the rate of neurite growth can also be directed with physical cues such as the size, inter-distance, and anisotropy of nanostructures.^6,8,13^

The cleft distance, a quantitative measure of vertical coupling between the cell membrane and surface, has been shown to depend on the underlying substrate terrain. Cui and coworkers analyzed *via* a high resolution focused ion beam scanning electron microscope (FIB-SEM) a significantly decreased cleft distance with nanopillars compared with nanopores and flat features (Fig. 1c and d).^14^ Reducing the cleft distance has important implications for electrochemical neural interfaces in terms of the seal resistance between the cell and electrode. Junctions formed after cells were cultured on electrodes with vertical nanostructures yielded seal resistances that range between tens to hundreds of MΩ, which are multi-fold greater than the seal resistances of cellular contacts with planar electrodes of several MΩ.^15,16^ Significantly improved extracellular neural stimulation and recording could be achieved as a result. Intracellular monitoring of electric activity, typically conducted by using patch-clamp devices, could also be carried out with electrodes after local electro- or opto-poration of the neuronal membrane made possible by the nanoprotrusions on electrodes.^17^

Expected to reduce foreign body response of implanted neural probes, control of surface topography can help develop selective interfaces with neurons when exposed to the heterogeneous neuron-glia population of the brain. High neuron-to-glia coverage ratios have been achieved on dealloyed nanoporous gold^18,19^ or on arrays of vertical nanowires^20^ compared to the planar control. Reduced non-neuronal cell coverage was feature size-dependent due to the lack of the substrate surface area available on smaller surface features for focal adhesion (Fig. 1b). A greater surface area is required for glial cells attachment than for neurons. Nanostructures could be fabricated on less rigid organic materials that are more suitable for *in vivo* neural probe design to yield similar results of suppressed astrocyte adhesion despite enhanced neurite outgrowth.^21^

The topographical effect on seamless integration can increase seal resistance and induce low gliosis, which is ultimately advantageous for effective stimulation and monitoring of the nervous system and preservation of intact neural-electrode contact over time.

#### 2.1.2 Effect of surface chemistry of substrates

Artificial substrates can establish cellular adhesion by tailoring their surface chemistry with materials that can form interactions with the extracellular cell matrix (ECM). These materials include cell adhesion molecules (CAMs) in neurons, representative of which are homophilic cadherins or heterophilic integrins, which can serve as anchoring sites during cell–cell or cell–matrix interactions.^22,23^ Short peptide sequences, such as RGD and YIGSR, which naturally occur in ECM proteins and are responsible for binding to receptors, have been modified on hydrogels or polymers to create synthetic ECM mimics. Comprehensive reviews have covered the host of materials for cellular adhesion and neural interfacing.^24–26^ Recent developments in surface modification for cellular adhesion are geared towards enhancing the long-term stability of modified surfaces and prevention of a foreign body reaction.

The surface charge and chemistry of the neuronal membrane have also been utilized to form tight junctions at the neuron–substrate interface. The overall negative charge of the plasma membrane, due to a plethora of negatively charged phospholipids, allows for cell adhesion *via* electrostatic interactions with positively charged molecules such as polymers containing amine functional groups. Examples of such polymers include poly-d-lysine and poly-l-lysine. Poly-dl-ornithine has also demonstrated neuron adhesion on artificial scaffolds.^11^ Surfaces modified with other positively charged molecules such as (3-aminopropyl)triethoxysilane (APTES) have shown considerable growth of neurons with greater seal resistance than that of poly-d-lysine.^3^ Molecular chirality plays a role in either improved interaction with ECM proteins (l-form) or resistance towards protease digestion (d-form). Surface modification with a lipid bilayer is another strategy to mimic the natural microenvironment of the plasma membrane such that proteins can maintain their structural complexity and mobility for receptor clustering to enable transmembrane proteins to perform their biological function.^27^

**Fig. 2:**
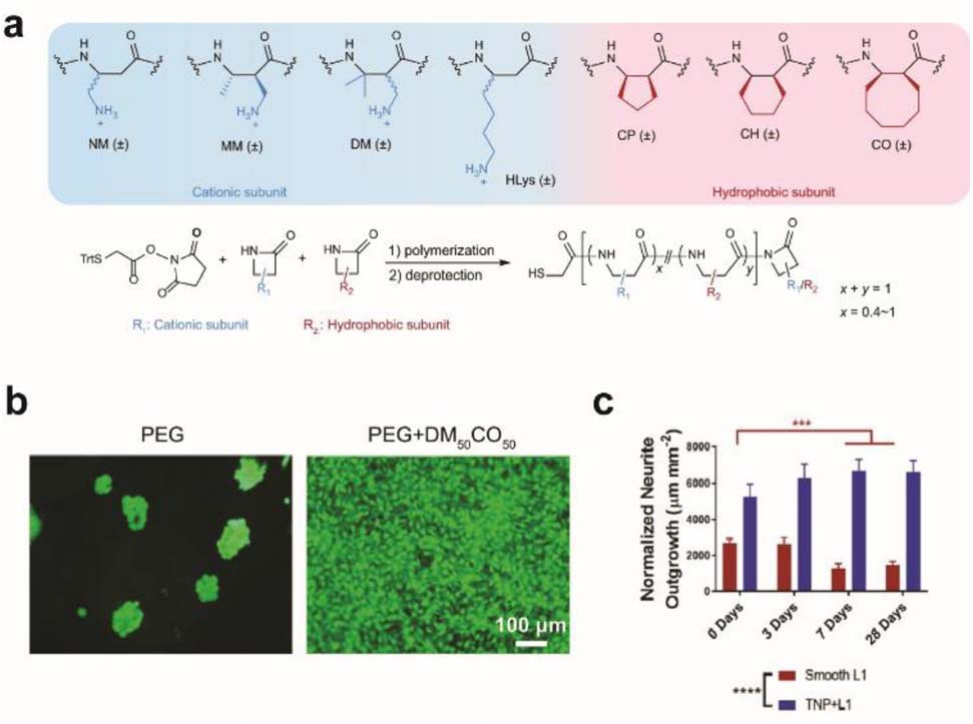
Effect of surface modification on cellular growth and *in vivo* implantation. (a) Beta-amino acid subunits to compose synthetic polymers. All subunits are racemic and the synthesized polymers are heterochiral. General synthesis of polymers with a chain length of 20 amino acid residues. (b) Live/dead assay for preosteoblast cells seeded on a bare PEG hydrogel and DM_50_CO_50_-modified PEG hydrogel for 2 days. Adapted from ref. 28 with permission from Nature Publishing Group, copyright 2021. (c) Bar graph quantifying normalized neurite outgrowth. Mean ± s.d. (*n* = 12 trials). Statistical significance was determined with a two-way ANOVA and Tukey's post hoc **p < 0.01 ***p < 0.001 ****p < 0.0001. Adapted from ref. 30 with permission from John Wiley and Sons, copyright 2021.

Synthetic or multiplexed approaches have been developed to modify surfaces for interfacing with cells. Cell adhesive polymers with enhanced stability were synthesized to mimic the cell adhesion functions and mechanisms of RGD and KRSR peptides (Fig. 2a and b).^28^ Improvement in long-term interfacing with cells *in vivo* was demonstrated in comparison to commercial coating materials for implants. In another study, a soft polymer was conjugated with two types of biomolecules where one enhances anti-inflammatory response with neuroprotective properties and the other prevents non-specific protein adsorption. Together a reduction in glial scar formation occurred during 7 days of implantation.^29^ A combination of chemical functionalization and nanotopography has synergistically improved interfacing with neurons. Proteins that promote adhesion between neurons, such as neural adhesion molecule L1, can be used to modify substrates to form neuron-specific interactions. L1 and nanoparticles have been dually coated on electrode surfaces to attenuate gliosis and enable recording of neural activity up to 4 weeks during *in vivo* implantation (Fig. 2c).^30^

Maintaining operational stability due to decreased foreign body response is an active area of research in the field of neural interfaces. However, other factors leading to the breakdown of the interface, such as migration of neurons away from the probe, should also be studied in order to achieve long-term communication with neurons.

### 2.2 Integration with the specific area of the neuronal membrane

#### 2.2.1 Non-synaptic interface

**Fig. 3:**
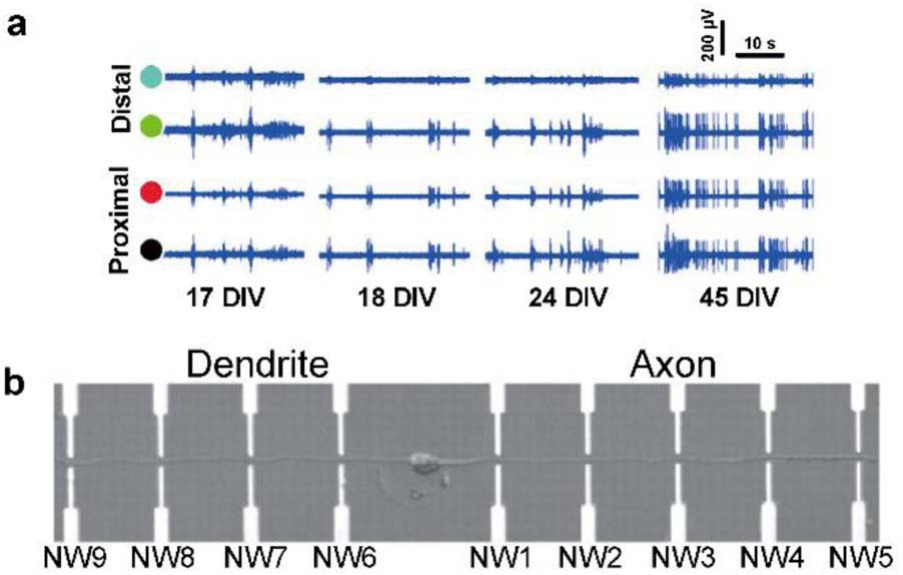
Observations of intracellular signal propagation by targeting neurites (axons or dendrites). (a) Recording of activity by two distal (green and blue circles) and two proximal (black and red circles) microelectrodes from the position of laser-induced dissection of axons. The activity recovery was observed on distal electrodes for several weeks. Adapted from ref. 32 with permission from the Royal Society of Chemistry, copyright 2015. (b) Single neuron cultured on a nanowire (NW) transistor array. Axon and dendrite are aligned oppositely by micropatterned poly-l-lysine. Adapted from ref. 33 with permission from the American Association for the Advancement of Science, copyright 2006.

This section focuses on research concerning the formation of specific interfaces between electrodes and subcellular regions of neurons, namely the cell body (soma) and neurites (dendrites and axon). The distinct size and morphology of subcellular structures can be utilized as differentiating factors to facilitate the alignment of neurons onto electrodes. Neurons could be compartmentalized into somata and neurites based on their cellular dimensions using microfluidic chambers that contain reservoirs connected by microchannels. Further separation of neurites into axons and dendrites can be possible by adjusting the length or height of the microchannels.^31^ Axon regeneration was monitored by measuring the neural activity of cultured neurons of the central nervous system (CNS) after axon injury through a microfluidic culture platform integrated with a microelectrode array (Fig. 3a).^32^ In another approach, substrates with micropatterned cell adhesion molecules displayed guided growth of neurons in such a way that axons or dendrites are positioned over sensing electrodes. In this manner, one to one contact between a neuron and nanowire transistor could be achieved without overlapping with other neurons on optimized micropatterned designs of poly-lysine on the nanowire transistor array (Fig. 3b). Through this platform, electrical stimulation as well as action potential recording could be achieved in targeted neurite regions, which was used to differentiate the rate of intracellular electrical signal propagation between dendrites and axons.^33^ The neuron-electrode interface formed between specific subcellular regions of neurons shows potential for neuro-modulation and neurosensing with high spatial control.

#### 2.2.2 Synaptic interfaces

**Fig. 4:**
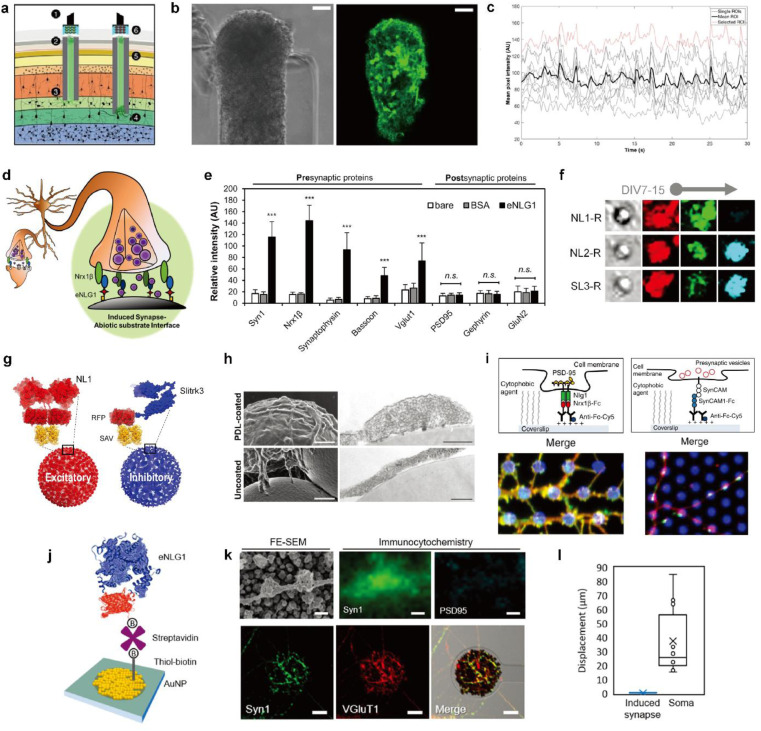
Biohybrid strategies for the formation of the synaptic interface. (a) Schematic illustration of the implanted μTENN which is able to transmit signals with neurons bidirectionally into the brain. Optogenetically active μTENNs as transplantable input/output channels. Inputs: an LED array (1) optically stimulates a unidirectional, channelrhodopsin-positive μTENN (2) to activate layer IV neurons (3). Outputs: layer V neurons (4) are connected *via* synapses to bidirectional μTENN (5) and relayed neuronal activity is recorded by a photodiode array on the brain surface (6). (b) Phase image of a part of bidirectional GCaMP+ μTENN before implantation in the rodent cortex. Scale bar: 50 μm (left). Multiphoton image of the same μTENN obtained immediately after implantation. Scale bar: 20 μm (right). (c) *in vivo* recording of calcium concentration changes with (b). Each red and grey trace corresponds to the time course fluorescence intensity of each region of interest (ROI) and mean trace is marked black. Adapted from ref. 34 with permission from the American Association for the Advancement of Science, copyright 2021. (d) Conceptual schematic of the induced presynapse interface. Presynaptic differentiation occurs at the contact between the neuronal membrane and the presynapse inducing protein functionalized substrate. (e) Fluorescence analysis of pre- and post-synaptic proteins under each bead condition. Intensities are compared between each bead condition. (37 < *n* < 68 for each condition. Statistical significance is indicated by n.s. for *p* > 0.05 and *** for *p* < 0.001). Adapted from ref. 37 with permission from the American Chemical Society, copyright 2019. (f) Neuroglin1 (NL1-R), neuroligin2 (NL2-R) and Slitrks3 (SL3-R) coated beads induce the formation of glutamatergic and inhibitory presynaptic boutons, respectively. Representative confocal images of induced hemisynapses immunostained with synapsin1 (Syn1, red), vesicular glutamate transporter 1 (VGluT1, green), and vesicular γ-aminobutyric acid (GABA) transporter (VGAT, cyan). (g) Structures of genetically engineered synapse-inducing proteins modified on beads (Red: NL1-R and blue: SL3-R), which can induce excitatory or inhibitory synapses. Adapted from ref. 36 with permission from Springer Nature, copyright 2016. (h) Poly-d-lysine (PDL)-beads induce the formation of synaptic vesicle complexes on axons. SEM (left) and transmission electron microscopy (TEM) (right) images of neurons cocultured for 24 h with PDL-beads (top) or uncoated beads (bottom). Scale bars: 1 μm (SEM) and 250 nm (TEM). Adapted from ref. 45 with permission from Society for Neuroscience, copyright 2009. (i) Molecular interactions at a single (1) NLG1/Nrx1β contact, leading to the recruitment of postsynaptic proteins, including PSD-95 (yellow), and (2) SynCAM1/SynCAM contact, leading to the recruitment of presynaptic vesicles (red). Immunostained images of synaptic protein recruitment at coated micropatterns. Left image shows the Nrx1β-Fc coated dots (blue) and enrichment of NLG1 (red) and PSD-95 (green). Right image shows SynCAM1-Fc boated dots (blue) and accumulation of SynCAM1 (red) and synapsin1 (green). Adapted from ref. 40 with permission from Nature Publishing Group, copyright 2013. (j) Scheme of eNL1 immobilized on an AuNP electrode. Structure of genetically engineered neuroligin-1, eNLG1 reconstructed after NLG1 (PDB ID, 3BIW) and TagRFP (PDB ID, 3NED). (k) Presynaptic differentiation of primary hippocampal neurons induced by the eNLG1 electrode. FE-SEM and confocal image of neurites immunostained with Syn1 (green) and PSD95 (cyan) on the eNLG1 electrode. Scale bar: 500 nm (top). Confocal images of neurites immunostained with Syn1 (green) and VGluT1 (red) on the eNLG1 electrode. Scale bar: 10 μm (bottom). (l) Displacement of somas and induced presynapses on the eNLG1 electrode observed over 7 days. Adapted from ref. 38 with permission from the American Chemical Society, copyright 2021.

Synaptic interfaces are one of the latest research trends in the seamless integration of neurons and electrodes. Studies have demonstrated synaptic connection of neurons in the cortex and optical neural probe by implanting a neuron-embedded hydrogel microcolumn into the brain.^34,35^ This platform, called the microtissue engineered neural network (μTENN) (Fig. 4a), enables optogenetic neural stimulation and *in vitro* optical recording of neural signals through multiple synapses formed in a single probe. The μTENN communicates with neurons in the brain through axon tracts and can transmit a relatively large number of signals through synapses formed with multiple neurons (Fig. 4b and c).

However, the random formation of synapses limits the ability to control the type, number, and location of the synapses. Therefore, efforts to develop methods to control synaptic connectivity are necessary to create a more sophisticated electrochemical neural interface using synaptic transmission. A new strategy for neural interfacing has been proposed to apply surface modification to electrodes that distinguish between types of synapses formed.^36–38^ The methodology involves inducing type-specific hemi-synapse formation on electrodes functionalized with synapse-inducing proteins. Here, the electrode replaces one part of the presynaptic-postsynaptic membranes. The following will explore strategies required to create an electrochemical synaptic interface.

One mechanism of synapse formation involves initial protein recognition and binding followed by downstream cellular processes leading to a mature synapse.^39^ Synapse-inducing molecules could be chemically functionalized on materials to trigger intracellular processes involved in synapse formation to form nascent hemi-synapses. We propose to name this type of synapses on non-neuronal substrates ‘Janus synapses’. Janus synapses are asymmetric contacts between pre-or postsynaptic organization of neurons and non-neuronal substrates (Fig. 4d).^37,38,40,41^ Diverse synapse-inducing molecules have been functionalized on different substrates, including non-neuronal cells,^42^ solid supported lipid bilayers (SLB),^43^ microbeads,^36,37,41^ glass,^40,44^ and electrodes^38^ (Fig. 4d–l).

One class of synapse-inducing molecules comprises polymers or compounds with positively charged amines. Microbeads modified with polymers^45,46^ and phospholipids^47^ with primary amine moieties have shown to induce presynaptic boutons, such as poly-d-lysine and phosphatidylethanolamine, whereas molecules with tertiary amines were unsuccessful. However, other primary amine phospholipids, such as phosphatidylserine, were interestingly non-synaptogenic. Molecular and physical properties governing synaptogenesis for these modified surfaces and the underlying mechanism are not yet clear, but may provide key information for the design and successful formation of induced synaptic interfaces between neurons and artificial substrates.^47^

In contrast to the abovementioned synthetic synapse inducing molecules, there are transmembrane proteins found in the synaptic cleft, which are responsible for synapse formation, differentiation, and maintenance, called synaptic adhesion molecules (SAMs).^48^ As one type of SAM, the neuroligin (NL) and neurexin (NRX) family of proteins have particularly garnered interest due to their heterophilic binding properties that enable the selective formation of pre- or post-synaptic specializations depending on whether NL or NRX is immobilized on the substrate, respectively. Furthermore, NL isoforms, such as neuroligin-1 (NL1) or neuroligin-2 (NL2), can direct the formation of glutamatergic or dopaminergic presynaptic organizations (Fig. 4f).^36,49^ Kim *et al.* reported that the age of neurons at the initial contact with the substrate is also a determining factor for the synapse type for some NL isoforms.^36^ Other types of SAMs used in the construction of Janus synapses include Slitks3-PTPδ (Fig. 4f and g)^36^ and SynCAM1 (Fig. 4i).^40^ Many types of SAMs have been identified but have not yet been tailored for immobilization on biological and non-biological substrates. Doing so will help expand the library of Janus synaptic interfaces.

While studies on synapses formed between neurons and artificial substrates were mainly focused on understanding synaptogenesis, Janus synapses have also been investigated as a potential interfacing approach for electrochemical neural interfaces. As a model system, NL1 was widely used in the study of Janus synaptic interfaces. Genetic mutation studies helped identify the ectodomain of NL1 responsible for synapse induction.^50,51^ A modified form of the NL1 protein could be engineered that is better suited for immobilization on non-biological substrates using diverse surface chemistries. Most recently, Janus synapses were formed on electrodes by Chung and coworkers (Fig. 4j–l).^38^ This is the first example of Janus synapse-electrode interfaces that selectively target the synaptic organization of living neurons *via* surface chemistry. Significantly, the artificial synaptic interface demonstrates membrane anchoring, which facilitates robust adhesion to the interacting area of neurites. Jeon *et al.* showed that artificial induced presynapses on NL1 modified electrodes did not show spatial displacement, and in contrast neuronal soma on the poly-d-lysine and laminin coated insulation layer migrated with an average cell movement of 37.4 μm over 7 DIV (Fig. 4l).^38^ Taken together, the neuron-electrode synapses can be translated to the electrochemical neural interface with significant implications. The properties of synapses can be applied which are advantageous for electrochemical neural interfaces, such as short intermembrane distance, synapse-type specificity, and synaptic plasticity. Most importantly, Janus synapses may enable the formation of single neuron-single electrode or single neuron-multiple electrode contacts.

### 2.3 Other important considerations for seamless integration of neural probes

#### 2.3.1 Coating of antifouling agents

Research on antifouling is crucial for developing effective neural interfaces for *in vivo* applications. Electrode fouling is largely classified into biofouling and electrochemical fouling. Non-specific adhesion of undesired biomolecules on the electrode surface hinders mass transport of analytes to the electrode. Electrode fouling results in passivation of active sites on the electrode surface which decreases sensor performance. This section will focus on improving the antifouling properties of neural electrodes through surface modifications.

In addition to conventional surface functionalization materials to prevent biofouling, such as hydrophilic poly(ethylene glycol) (PEG) films,^52^ new materials are continuously developed. The following considerations are taken when selecting surface modifying molecules to reduce biofouling: (1) size exclusion and (2) hydrophilicity or electrostatic repulsion. This section explores pioneering studies regarding these issues.

**Fig. 5:**
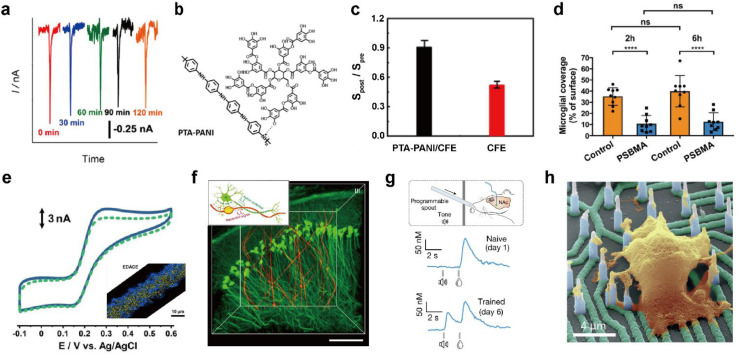
Representative designing considerations for seamless neural probes. (a) *In vivo* monitoring of dissolved oxygen in the hippocampus of the rat brain with an electrografted silica nanoporous membrane (SNM) modified column-shaped carbon fiber microelectrode (CFME) every 30 minutes for 2 h. Adapted from ref. 53 with permission from the American Chemical Society, copyright 2019. (b) Chemical structure of polytannic acid doped nanoporous conductive polyaniline (PTA-PANI). (c) The ratios of sensitivities by postcalibration of the PTA-PANI-coated CFME (black column) and bare CFME (red column) to that by precalibration in artificial cerebral spinal fluid before and after implant in the rat brain for 2 h. Adapted from ref. 54 with permission from the American Chemical Society, copyright 2019. (d) Microglial surface coverage of poly(sulfobetaine methacrylate) (PSBMA) coated probe (blue bars) and non-coated probe (orange bars) surfaces (Two-way ANOVA and Tukey's post-hoc tests; ****p < 0.0001 for both 2 and 6 h). Adapted from ref. 56 with permission from Wiley, copyright 2020. (e) Cyclic voltammogram obtained in 10 μM dopamine (DA) solution at the electrodeposited graphene oxide microband-CFME before (solid curve) and after (dotted curve) immersion into 20 mg mL^−1^ BSA for 2 h. The inset shows energy dispersive X-ray analysis (EDX) of the electrodeposited graphene oxide microband-CFME. Au and graphene oxide are respectively colored yellow and blue. Adapted from ref. 58 with permission from John Wiley and Sons, copyright 2021. (f) Three-dimensional reconstructed images of neuron-like electronics (NeuE)-neural tissue interface 3 months after implantation. (neuron (green) and NeuE (red)) Scale bar: 100 μm. Inset shows the structural similarity between neurons and NeuE. Adapted from ref. 66 with permission from Springer Nature, copyright 2019. (g) Experimental scheme for a Pavlovian reward learning task in freely moving mice using NeuroString (top). Exemplar time-aligned DA signals from a mouse on day 1 (middle) and day 6 (bottom). Adapted from ref. 67 with permission from Springer Nature, copyright 2022. (h) False-colored SEM image of cultured neuron on vertical nanoelectrode array. Scale bar: 4 μm. Adapted from ref. 69 with permission from the American Chemical Society, copyright 2017.

Nanoporous films with pore sizes smaller than that of foulants can block their access to active sites of electrodes. In one study, a nanoporous membrane deposited carbon fiber microelectrode (CFME) was used to prevent the passage of proteins while allowing diffusion of small molecules such as oxygen.^53^ Thus the prepared electrodes could monitor oxygen levels when implanted in the mammalian brain for two hours (Fig. 5a).

Electrode functionalization with hydrophilic functional groups helps form a hydration layer that weakens interaction between the electrode and foulants to minimize biofouling. Biofouling was reduced all the while increasing electrochemical performance through nanoporous conductive polymer coating.^54^ Polytannic acid (PTA)-doped nanoporous conductive polyaniline (PANI) was electrochemically polymerized on the CFME (Fig. 5b). The large number of the phenolic hydroxyl groups and nanoporous structures present in PTA-PANI increased the hydrophilicity and alleviated non-specific binding of proteins. The PTA-PANI coated CFME showed almost similar sensitivity before and after *in vivo* dopamine measurement (Fig. 5c). Polymers with alternating pendant groups of positive and negative charges, such as in zwitterionic polymers or zwitterionic peptides, accumulates a strong hydration layer near the electrode surface by ionic solvation.^55^ Recently, *in vivo* bio-integration of neural probes for long-term implantation by coating zwitterionic materials on electrode surfaces has been reported (Fig. 5d).^56,57^ Another strategy to prevent biofouling is surface functionalization to enhance electrostatic repulsion between surface bound molecules and biomolecules. Electrode functionalization with materials, such as graphene oxide with various hydrophilic functional groups including hydroxyl, epoxy, and carboxyl groups, enables *in vivo* monitoring with decreased biofouling. The antifouling effect of electrodeposited graphene oxide towards biomolecules was characterized using cyclic voltammetry before and after exposure to bovine serum albumin (BSA) (Fig. 5e). The antifouling electrode was immobilized with Ca^2+^ binding ligands to measure extracellular Ca^2+^ concentration in the brain *in vivo*.^58^

Unlike biofouling in which the causative molecules are exogenous, electrochemical fouling occurs when the chemical species generated by electrochemical reactions cover the electrode surface. In particular, monoamine neurotransmitters could be reduced or oxidized to highly reactive species near the electrode. These products polymerize easily under physiological conditions and the resulting polymer film insulates the electrode. Representative molecules are dopamine and 5-hydroxytryptamine (5-HT; serotonin). To reduce such electrochemical fouling, surface modification of the electrode with coatings such as functionalized nanodiamonds has been attempted.^59,60^

#### 2.3.2 Flexibility

Several electrodes are often fabricated as an array type to manipulate or analyze neural networks. Flexible materials with similar mechanical properties to neurons have been used as encapsulating layers on neural probes in order to mitigate immune response and uniformly integrate with brain tissues.^61^ This perspective does not cover the trends in flexible neuroelectronics, so please refer to other in-depth review papers.^62–65^ Herein, we will explore representative flexible neural probes for *in vivo* electrical and electrochemical neural recording. Yang *et al.* have developed bioinspired neural electrodes for chronic *in vivo* recording of neuronal electrical signals by mimicking the shape and physical properties of neurons.^66^ A key design parameter of these neuron-like electronics (NeuE) is their similarity in size to neurons (Fig. 5f), which results in comparable bending stiffness to that of axons. Foreign body responses such as glial encapsulation was reduced and seamless integration with the brain allowed recording of electrophysiological signals at a level of fewer than 5 cells for up to 3 months. Furthermore, *in vivo* neurochemical recording could be performed with flexible and stretchable electrodes for long-term, selective, and sensitive detection of neurotransmitters.^67,68^ Li *et al.* developed a tissue-like flexible monoamine sensor called NeuroString which can operate in both the brain and the gut (Fig. 5g).^67^ The sensor consists of an Fe_3_O_4_-graphene electrode embedded in polystyrene elastomer to monitor the dopamine concentration of an optically stimulated ventral segmental area (VTA) in real-time for 16 weeks using fast-scan cyclic voltammetry (FSCV).

#### 2.3.3 Electrode size and high density of electrodes

Most long-term attempts to record neural activity *in vivo* using neural probes still fail to achieve spatial resolutions below the level of a single cell because the electrode size is comparable to or larger than that of neurons. To precisely control and understand information processing of the nervous system, the size of electrodes must be smaller. Many researchers have strived to reduce the electrode sizes of highly integrated electrode arrays. The action potentials of single cultured neurons were measured *in vitro* with individually wired vertical nanowire arrays (Fig. 5h), which have an electrode diameter and inter-electrode spacing of a few hundred nanometers.^69^ The electrical signals of neurons were observed with amplitudes of 0.1–99 mV which were comparable to the signal amplitude of intracellular recordings. To the best of our knowledge, nano-protrusion arrays integrated on flexible substrates have not yet been applied to neural interfacing. If nanoelectrode arrays can be grafted onto flexible materials, neural signals in the brain can be monitored at the level of a single cell or less, allowing for more precise mapping and analysis of neural activity.

## 3. Electrochemical monitoring of neurochemicals with a focus on surface modification

This section highlights important electrochemical techniques and electrode modifications required to monitor neurochemicals that regulate neural activity. Key technical requirements must be met for neurosensing *in vivo*. High selectivity towards the target is required for detection from a complex neurochemical mixture. High temporal resolution can provide information on neural signalling at various time scales, the most elusive of which is synaptic transmission occurring in sub-millisecond time frames.^70^ Signal sensitivity is important to detect small chemical fluctuations in the local environment of neurons that have implications for neural signalling and pharmaceutical research.^71^ The stability of the signal must be maintained for the duration of neurochemical monitoring. Electrochemical techniques in themselves fall short of meeting the above-mentioned criteria, and therefore electrodes are immobilized with high-performance molecular recognition elements that can selectively bind to neurochemicals.

### 3.1 Electrochemical techniques for neurochemical sensing

This section introduces representative electrochemical techniques that can be used at electrochemical neural interfaces.

#### 3.1.1 Amperometry

Amperometry is carried out by measuring the oxidation or reduction current of neurochemicals by applying a constant potential. This technique has higher temporal resolution than other electrochemical techniques such as voltammetry or potentiometry, which makes it possible to detect changes in the analyte on sub-millisecond timescales. With the use of a microelectrode or nanoelectrode, it is also possible to quantify the number of molecules at the zepto-molar level with a high signal-to-noise ratio. Therefore, not only changes in extracellular concentration but also quantal release of neurotransmitters due to vesicular exocytosis can be observed in neurons and neuroendocrine cells.

**Fig. 6:**
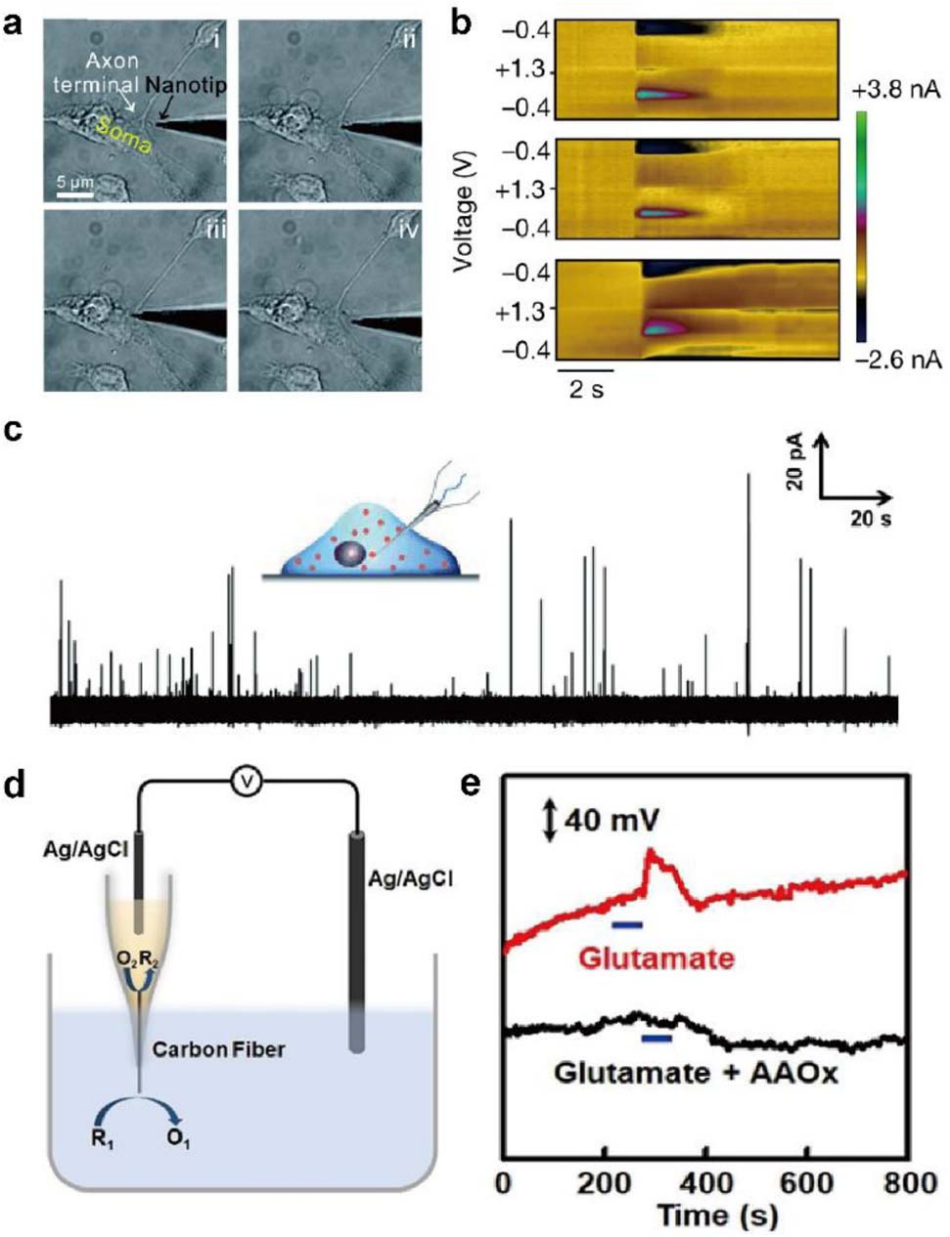
Representative electrochemical techniques for neural interfaces in recent studies. (a) Microscopy images of the insertion and withdrawal process of a conical carbon fiber nanoelectrode (CFNE) in the synaptic cleft in chronological order. Adapted from ref. 74 with permission from the Royal Society of Chemistry, copyright 2020. (b) *in vivo* FSCV detection of dopamine in the nucleus accumbens by a three-channel NeuroString sensor during optogenetic stimulation (20 Hz with 15 pulses) of dopaminergic neurons in the ventral tegmental area. Adapted from ref. 67 with permission from Springer Nature, copyright 2022. (c) Amperometric trace for a nanotip conical CFME placed inside a PC12 cell. Adapted from ref. 75 with permission from John Wiley and Sons, copyright 2015. (d) Schematic illustration of the potentiometric sensor consisting of a voltmeter, an Ag/AgCl reference electrode, and a closed bipolar CFME which can oxidize ascorbate spontaneously. (e) *In vivo* potentiometric sensing of the concentration dynamics of ascorbate in the striatum upon locally injecting 100 μM glutamate (red curve) or a mixture of 100 μM glutamate and ascorbate oxidase (40 units mL^−1^) at a rate of 2 μL min^−1^ for 60 s (black curve). Adapted from ref. 88 with permission from John Wiley and Sons, copyright 2020.

Deciphering the cellular mechanisms underlying neural signal transmission has been a primary research goal for many researchers. Huang, Amatore, and their colleagues inserted nanoelectrodes within the neuronal synaptic cleft to study inter-neuron communication *via* neurotransmitters (Fig. 6a).^72–74^ The fabrication of neural electrodes with size dimensions similar to that of the synaptic cleft of approximately 20 nm is crucial. Using nanoelectrodes, the quantal release of neurotransmitters at different types of synapses was detected in real-time to help understand the mechanisms of synaptic exocytosis and the effects of pharmaceutical drugs.^72–74^ Since nanoelectrodes cause relatively small damage to cells when penetrating the cell membrane, their less invasive nature has enabled real-time amperometric analysis of chemicals inside the cell body (Fig. 6c). Ewing and coworkers have demonstrated the usefulness of intracellular vesicular electrochemical cytometry for studying exocytosis mechanisms and quantifying intracellular chemicals in various cell types.^75–79^

However, a critical weakness of amperometry is its lack of chemical selectivity, limiting the operation of *in vivo* amperometric sensors. Developments in many real-time *in vivo* amperometric neurochemical sensors with chemical selectivity are expected with techniques discussed in Section 3.2.

#### 3.1.2 Fast-scan cyclic voltammetry (FSCV)

FSCV is a widely used electrochemical technique for real-time sensing of neurochemicals with sub-second temporal resolution (Fig. 6b). Changes in neurochemical concentration can be analyzed by subtracting the background current from a cyclic voltammogram obtained in the presence of neurochemicals. Large background current results from sweeping the potential at a high scan rate of more than 100 V s^−1^. Changes in the electrode surface and environment surrounding the electrode can cause drifts in the background current, leading to artefacts from background subtraction. Efforts to diminish background subtraction artefacts have been suggested.^80–82^

Various waveforms are being developed to enhance selectivity, such as the triangle waveform used to measure dopamine, the Jackson waveform which is a conventional waveform for serotonin detection, and the multiple scan rate waveform used to measure methionine-enkephalin.^83^ Efforts are ongoing to reduce electrode fouling with FSCV, as it is widely used for *in vivo* neurochemical monitoring due to its high chemical selectivity.^84–86^ A study developed a new FSCV waveform to mitigate electrode fouling during serotonin detection.^84^ Furthermore, the lifetime of an electrochemical dopamine sensor could be expanded through potential cycling, which cleans the electrode surface and prevents electrode fouling when implanted in the brain.^86^

The detection of electroinactive neurotransmitters has been attempted with FSCV. Enzyme-catalysed detection of acetylcholine was possible at high potential sweep rates of 400 V s^−1^ by coating acetylcholine esterase and choline oxidase on a CFME. The conversion of acetylcholine to hydrogen peroxide by enzymes and subsequent electrochemical oxidation of hydrogen peroxide at the electrode was observed.^87^

#### 3.1.3 Potentiometry

Along with electrochemical techniques described above, potentiometry can also be employed for neurochemical detection. Potentiometric devices include ion selective sensors and redox potentiometric sensors. Ion concentrations can be monitored by measuring the potential difference determined by the concentration of ions bound to ionophores or ligands in ion selective electrodes. Tian and coworkers recently observed extracellular Ca^2+^ changes during an ischemia-reperfusion process in seven brain regions of a freely moving rat for 60 days with Ca^2+^ ligand modified electrodes.^58^

In redox potentiometric sensors, the working principle is based on redox reactions. The open circuit potential (OCP) of the working electrode relative to the reference electrode is determined by the concentration ratio of the reduced and oxidized forms on the electrode surface. The system must be in chemical equilibrium for this relationship to apply and extract analyte concentration from the measured OCP. Galvanic closed bipolar electrodes could monitor ascorbate or hydrogen sulfide concentrations.^88,89^ At one end of the electrode, an electrochemical half reaction of the neurochemical selectively occurs through appropriate surface modifications. At the other end, the counter electrochemical reaction occurs spontaneously (Fig. 6d and e). A CFME potentiometric sensor modified with silver sulfide/silver nanoparticles can be operated *in vivo* by adopting a chemical reaction with a low solubility product.^90^ Due to the low current flow of several pico-amperes, the crosstalk issues of the OCP-based sensors with other electrochemical sensors can be avoided when operating two or more devices simultaneously.^91^

Potentiometric sensors, which observe changes in potential proportional to the logarithmic value of analyte concentration, are typically less sensitive than techniques that measure current changes in proportion to concentration, such as FSCV or amperometry. However, they can measure a wider range of analyte concentrations. Therefore, it is recommended to select a suitable electrochemical technique based on the sensing target and environment.

### 3.2 Immobilization of molecular recognition elements (MREs)

MREs are materials that can bind to a particular molecular target with high selectivity. They are used in neural interface research for selective electrochemical monitoring of both electroinactive and electroactive neurochemicals. While a small subset of neurochemicals contains electroactive functional groups (such as catechol) that can directly transfer electrons to and from an electrode at mild electrode potentials and physiological pH, the majority of neurochemicals are electroinactive and require MREs to facilitate electrochemical detection. Moreover, the extracellular environment has multiple electroactive species with overlapping redox potentials. Signal selectivity can be improved with MREs during electrochemical monitoring of electroinactive species.^92^

MREs can be classified based on the method of electrochemical signal generation. A common type of MRE is enzymes which catalyze the reduction or oxidation reaction of molecular targets. Non-catalytic MREs, such as aptamers, create electrochemical signals through structural changes in the recognition element, which is caused by binding with the molecular target. In this section, we explore recent developments in the two representative groups of enzymatic and aptamer sensors tailored towards *in vivo* monitoring. Other recognition elements for neurochemical monitoring, such as metal oxide electrocatalysts, molecularly imprinted polymers (MIPs) and ionophores, have been detailed in other reviews.^93–96^

Typical electrode functionalization with MREs is carried out by electrochemical or chemical methods, such as electrografting, electro-polymerization, chemical oxidation, and formation of self-assembled monolayers. Electrodes are often pretreated to produce orthogonal handles for MRE conjugation on their surface.^97–99^ The subsequent immobilization of MREs can occur *via* electrostatic interactions, crosslinking through linkages such as amide or imine, or physical entrapment during polymerization.^100^

This section focuses on the development of sensors to enable real-time communication with neural networks, in which enzymes and aptamers hold significant potential towards achieving this goal.

#### 3.2.1 Enzymes

As one of the most common recognition elements found in nature, enzymes permit electrochemical detection of electroinactive neurochemicals due to their highly selective substrate binding site and endogenous redox active site. To achieve chronic and real-time communication between neurons and electronic devices, enzymatic sensors must fulfil certain criteria including high temporal resolution, sensitivity and long-term stability. Each of these objectives is a long-standing goal of research in enzymatic biosensors for use in neural interfaces.

**Fig. 7:**
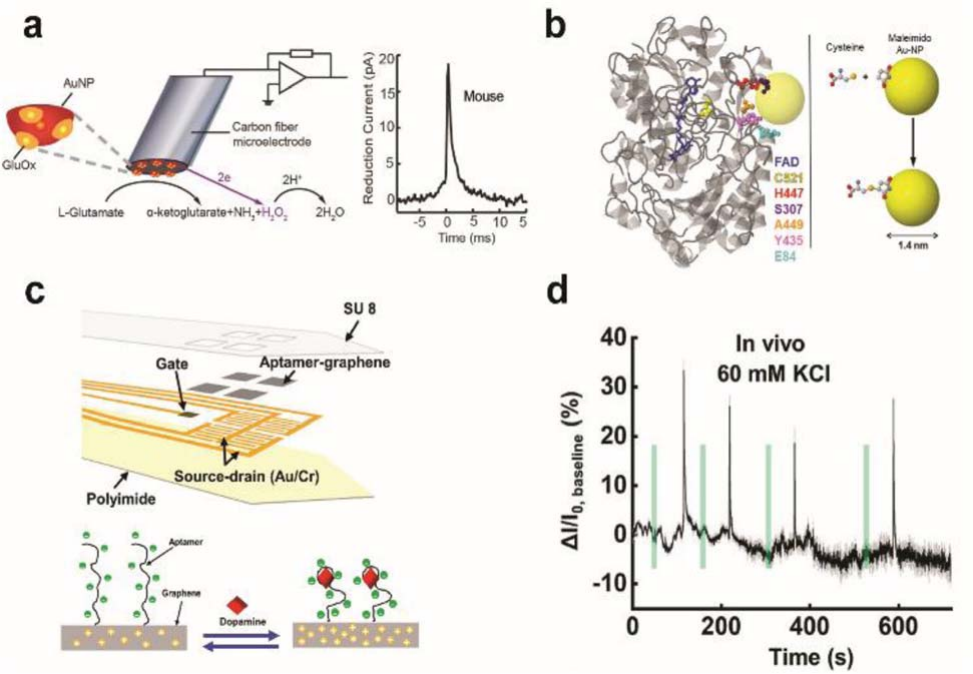
Design of enzymatic and apta-sensors for the detection of neurochemicals. (a) Schematic diagram of an ultra-fast enzyme sensor for glutamate detection. Carbon fiber microelectrode was modified with gold nanoparticles (red) and a thin layer of glutamate oxidase (yellow). Glutamate is electrochemically detected by the sensor *via* reduction of the reporter molecule hydrogen peroxide. Averaged amperometric current–time trace for individual exocytosis events detected from acute brain slices of the mouse is shown. Adapted from ref. 104 with permission from the American Chemical Society, copyright 2019. (b) Ribbon diagram of glucose oxidase monomer (from A. niger) with the FAD cofactor (blue) and immobilized gold nanoparticle (AuNP, yellow). Mutated amino acid residues are shown as space-filling models: cysteine (yellow), histidine (red), serine (purple), alanine (orange), tyrosine (pink), and glutamate (light blue). Binding of the AuNP to cysteine *via* covalent click chemistry is shown in the scheme. Adapted from ref. 111 with permission from the American Chemical Society, copyright 2011. (c) Schematic illustration of the implantable aptamer-graphene microtransistor probe design and conformation change of the aptamer upon dopamine binding. (d) Representative response trace for the neural probe implanted in striatum after local perfusion with 60 mM KCl. VDS = 100 mV and VG = 50 mV. Adapted from ref. 68 with permission from the American Chemical Society, copyright 2022.

Electrochemical enzymatic sensors with high temporal resolution comparable to the time scale of neural signalling is of significant importance, especially for monitoring synaptic transmission of electroinactive neurochemicals. Recently, first-generation enzyme electrodes have demonstrated the ability to detect quantal release of glutamate exocytosis down to the sub-millisecond resolution. Cans and coworkers have developed a new approach to minimize the distance that an electoactive enzyme product needs to diffuse *via* enzyme adsorption on gold nanoparticles to drastically improve the temporal resolution of enzyme sensors (Fig. 7a).^101^ Independently, Huang and coworkers demonstrated the ability to form an ultrafast glutamate biosensor by enzyme crosslinking on platinum nanoparticle coated nanowires.^102^ These enzymatic sensors have high selectivity and micromolar sensitivity for glutamate together with sub-millisecond temporal resolution to receive real-time synaptic neurochemical signals in neuron varicosities^103^ and brain slices.^104^ However, these enzymatic sensors are typically used short after preparation to maximize performance. Therefore, operational stability in long-term must be achieved for non-acute detection, which is a major hurdle against *in vivo* monitoring.

To date, implanted enzymatic sensors are only capable of measuring tonic changes in the concentrations of neurotransmitters such as d-serine,^105^ γ-aminobutyric acid (GABA),^106,107^ and glutamate.^108^ Typically, greater immobilization stability of enzymes can be achieved *via* cross-linking chemistry followed by encapsulation with a size exclusion barrier, including *meta*-phenylenediamine polymer, to prohibit the outward detachment of enzymes, albeit at the cost of sensitivity and temporal resolution.

First-generation sensors generate electrochemical signals through the redox reaction of electroactive by-products, such as hydrogen peroxide, at the electrode. A critical issue with these sensors is the continuous generation of these by-products, which is oftentimes harmful to neuron health. Additionally, they depend on the flux of co-substrates, most commonly oxygen, which affects the sensor performance under conditions where co-substrates are scarce. So far, *in vivo* monitoring was mostly demonstrated with first generation sensors with limitations. Developments in latter generations of enzymatic sensors for chronic *in vivo* monitoring are much needed.

To combat these first-generation shortcomings, second (mediated electron transfer, MET) and third (direct electron transfer, DET) generations of enzyme sensors have been developed to facilitate electron transfer to and from the electrode either through a redox mediator (MET) or through direct transfer (DET), respectively.^109,110^ Their operation principles reduce the formation of harmful enzymatic byproducts or the need for co-substrates. However, minimizing the electron tunnelling distance is a major bottleneck as enzyme active sites are typically deeply embedded in the protein architecture. Therefore, the control of electrode-enzyme (DET), electrode-mediator (MET) and enzyme-mediator (MET) distances is a crucial factor that can be tuned with surface modification techniques and genetic engineering of proteins when designing MET or DET based sensors. Holland *et al.* demonstrated DET between glucose oxidase and the electrode by placing a gold nanoparticle in close proximity to the enzyme active site *via* genetic engineering of enzyme amino acid residues (Fig. 7b).^111^ Apoenzyme-based sensors could establish proper protein orientation on the electrode surface to the minimize electron transfer (ET) distance between the enzyme active site and electrode.^112–115^ The reconstitution of apoenzymes with the enzyme cofactor that is tethered to the electrode surface created a well oriented enzyme layer, and the ET distance could be controlled by the lengths of various aliphatic linkers or short chain thiols connecting the co-factor to gold nanoparticles or redox mediator immobilized on the electrode. The oxygen dependence of the enzyme electrode was markedly reduced and the electron transfer rate was significantly increased; however, the stability of reconstituted apo-enzyme must be assessed for further applications *in vivo*. Furthermore, metal or semiconductor nanomaterials could be incorporated at the interface between the enzyme and electrode to enhance electron transfer kinetics, even across insulating organic layers typically used for enzyme immobilization on electrodes.^116,117^ Distance-independent electron transfer was obtained with metal-insulator-nanoparticle systems as a function of nanoparticle size and organic film thickness.^118^ Long-distance electron transfer could be achieved when nanoparticles were used to relay electrons between enzymes and electrodes.^117^ However, working demonstrations of these types of sensors for *in vivo* monitoring are limited.

The development of an immobilized enzyme layer that can undergo MET or DET in brain-implanted neural probes is the complex. In addition to neural probe design considerations for *in vivo* monitoring explored in Section 2.3, other factors such as mediator biocompatibility, mediator leaching, and stability of the well-oriented enzyme layer should be examined. Furthermore, there is a limited library of enzymes that can undergo facile MET or DET to catalyse neurochemicals. Perhaps this expansion of the enzyme library can be facilitated with research in protein engineering improving DET and MET for second and third-generation enzyme sensors.^119,120^

#### 3.2.2 Aptamers

Enzymes as a recognition element have drawbacks. Importantly, enzymes are susceptible to denaturation and loss of enzyme activity especially after immobilization on electrode surfaces. Maintaining the long-term performance and shelf life of enzyme electrodes can be challenging. Furthermore, enzymes are costly, making their commercialization difficult. Therefore, the development of non-enzymatic sensors has garnered much interest. Aptamers are an alternative type of MRE and have been used to develop sensors for *in vivo* monitoring of neurochemicals.^121^

Aptamers are short, single-stranded (<100 bp) nucleic acids that can specifically bind with a target molecule to undergo a conformational change. The selectivity of the aptamer for the target analyte is developed by a method called systematic evolution of ligands by exponential enrichment (SELEX). Aptamers that can recognize a selection of neurotransmitters, such as adenosine triphosphate (ATP), dopamine, and serotonin, have been reported.^122^

To generate an electrochemical readout signal, a redox probe is typically attached to the aptamer. Structural changes of the aptamer alter the electron transfer distance between the redox probe and electrode giving rise to a change in the electrochemical signal. As SELEX is commonly carried out in the homogeneous solution phase, care should be taken to maintain the binding affinity and chemical selectivity of aptamer sequences after functionalization with redox probes and immobilization on the electrode surface.

The design of electrochemical aptamer-based (EAB) sensors plays an important role in determining the sensing performance. Signal amplification could be achieved with the incorporation of various catalytic materials such as carbon-based nanomaterials, metal–organic frameworks (MOFs), gold nanoparticles, and polymers.^123^ Recently, EAB sensors were developed to monitor neurochemicals with temporal resolution down to a few seconds *in vivo*. Aptamer-immobilized field-effect transistor (FET) biosensors showed excellent capabilities for selective monitoring of neurotransmitter fluxes *in vivo* in real time. Nanoscale FET sensors with ultrathin (∼3 to 4 nm) In_2_O_3_ films were coupled with serotonin binding aptamers with femtomolar detection limits.^124^ Other FET sensors using aptamer-graphene microtransistors were able to detect in real-time dopamine release *in vivo* and with long-term stability of two weeks. Stability is attributed to pi–pi stacking between graphene and pyrene tagged dopamine aptamer (Fig. 7c and d).^68^ Furthermore, direct electrochemical conjugation of aptamers *via* electrografted catechol on a CFME has increased the stability of immobilized aptamers to facilitate long-term monitoring of dopamine dynamics in the brain.^92^ However, non-specific binding of matrix elements in biological fluids is a major issue in EAB sensors. Other issues include their lower sensitivity and selectivity than that of current state-of-the-art enzymatic sensors. These issues can be addressed by modulation of the aptamer structure to change their binding affinity (*e.g.* equilibrium dissociation constant, *K*_d_) and cross-reactivity to non-targets.

As EAB sensors heavily depend on selective binding of neurochemicals to a carefully tailored nucleic acid oligomer, it is difficult to compete with complex amino-acid based proteins such as enzymes or antibodies in terms of selectivity and sensitivity. However, due to their inherent stability and relative ease of wiring to the electrode, EAB sensors with further developments may contribute significantly to bidirectional communication *via* electrochemical neural interfacing.

## 4. Chemical stimulation of neural systems *via* iontronic devices

The above section deals with methodologies to obtain electrochemical readout of neuron activity. However, an equally important aspect of neural interfacing is the modulation of neurons. Electrical stimulation is possible by applying an electric field or injecting charge into the aqueous medium surrounding neurons, which is a large research area covered comprehensively by various review papers.^125–127^ An alternative neuro-modulation technique is chemical stimulation by manipulation of the local flux of chemical species affecting neuronal activity. As an emerging chemical delivery platform, iontronics garners much attention due to its operational compatibility with neural systems to form a closed aqueous circuit. The signal carriers traversing the two iontronic-neuronal systems are charged species, including neurochemicals.^128^

Iontronic devices have been primarily applied to mimic information processing in neuron networks. Typically composed of hydrogel-based information processing elements (*e.g.* diodes), these nonlinear elements can form a fully ionic aqueous circuit to imitate synaptic plasticity and dendritic integration.^129^ Meanwhile, the development of chemical delivery probes using iontronics is another endeavour with significant implications for research in neuromodulation.

A promising class of iontronic devices is the organic electronic ion pump (OEIP), an ionic chemical delivery system that uses ion-exchange membranes (IEMs).^129,130^ The working principle of the OEIP is electrophoretic ion transport driven by an electric field applied across a pair of electrodes that are situated in the source and target reservoirs, respectively. An IEM separates the source reservoir, which contains charged chemical species for delivery, and the target reservoir, where cells are located. IEM characteristics can determine the delivery performance and type of charged species of the device.^131^ Common IEMs found in the OEIP are charged polymers for charge-selectivity such as polyelectrolytes or organic conducting polymers incorporated in polyelectrolytes. The advantages of this type of chemical neuromodulation system include high precision and delivery of chemicals without solvent-flow or mechanical pressure.^132^ Control over the delivery amount and release dynamics is facilitated by careful manipulation of the potential bias with electrochemical techniques. Furthermore, only cells close to the device outlet are exposed to a high amount of released chemicals, which is beneficial for local stimulation of living cells. In addition, iontronic devices do not rely on a mechanical pump that transports chemicals along with solvent, which can cause unintended changes in the cellular environment. This is beneficial for the stimulation of biological systems with minimal perturbation of the target environment.^133^

However, an important operating condition for chemical stimulation with the OEIP is the application of a suitable electrochemical potential to prevent reactive oxygen species (ROS) generation. At low potentials within the ohmic region, the relationship between ionic current and voltage is linear. At higher operation potentials, the ionic current can be limited by the formation of a space-charge region at the IEM-solution interface due to concentration polarization, which particularly occurs at the inlet interface in OEIPs. Upon further increase in operation potential, the electric field in the space-charge region increases, which drives water-splitting reactions to form ROS that are harmful to cells.^134^ To prevent ROS formation, studies have attempted to increase the limiting current through strategies that alleviate mass transport limitations, such as expanding the surface area of the IEM-solution interface.^133^

While neuromodulation with these devices *in vivo*^130^ has been achieved, there are still improvements to be made in terms of temporal resolution, changes in pH, and controlling chemical leakages to mimic that of synapses. Efforts have been made in these respects.

Jonsson *et al.* enhanced the temporal resolution of ion delivery by decreasing the distance required for ion migration through a vertical ionic diode in their neurotransmitter delivery device.^135^ Charged chemicals could be released with temporal resolution of tens of milliseconds. Iontronic chemical delivery devices could be further optimized to approach the ultrafast temporal dynamics of neural signalling by taking into consideration the device geometry, especially by minimizing the thickness of the IEM.^136^ Changing the chemical composition of the IEM to improve the movement of chemical species to be delivered is another design consideration.^137^

**Fig. 8:**
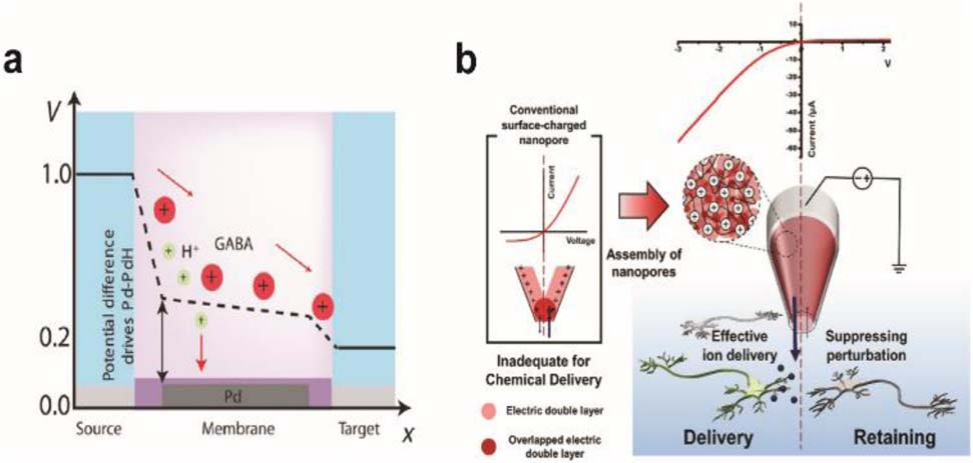
Chemical stimulation of neural systems *via* iontronic devices. (a) Potential profile across the palladium (Pd) based OEIP device. Proton and neurotransmitter GABA movement from the source to the target is shown. At certain potentials, the Pd electrode selectively absorbs protons to enable the passage of the drug to the target electrolyte. Adapted from ref. 140 with permission from the American Association for the Advancement of Science, copyright 2021. (b) Schematic illustration of the working principle for a PGF micropipette and comparison with a conventional surface-charged nanopore. Inverted ion current rectification enhances the outward flow in the PGF micropipette for chemical delivery. Adapted from ref. 141 with permission from the American Chemical Society, copyright 2021.

One side effect of iontronics-based chemical delivery is unintended changes in pH. During the delivery of cationic molecules, faster migration of protons due to their high mobility will lower the pH of the target environment that can be detrimental to cell health. Strakosas *et al.* minimized the interference of protons towards neurotransmitter GABA delivery through palladium electrodes placed under cationic exchange membrane layers that can electrochemically capture protons (Fig. 8a).^138^ Pd-based proton traps significantly enhanced the efficiency of GABA transport compared with devices without Pd electrodes while maintaining the pH environment of the solution.

Diffusive leakage is another major issue to be addressed, particularly for long-term implantations. Oh *et al.* fabricated a polyelectrolyte gel-filled micropipette with inverted ion rectification behaviour that effectively minimized unwanted chemical movement in the off state of chemical delivery (Fig. 8b). Applying a reverse bias potential reduced outward diffusive leakage of glutamate and repressed inward ion flux from and to the device, respectively.^139^

Overall, iontronics for neuro-modulation is a relatively new research field compared to electrochemical neuro-sensing. However, with further developments, it has the potential to play a significant role in achieving bidirectional communication in electrochemical neural interfaces using biomimetic information processing devices.

## 5. Perspective

Picking the brain in the metaphorical sense may soon become a relic of the bygone past, replaced by a more direct interrogation method to intercept and decipher messages that have silently coursed through the brain network. Physiochemical strategies for neural interfacing offer a promising path towards achieving seamless integration between neurons and neural probes, and have the potential to advance the development of brain-computer interfaces (BCIs). However, certain milestones must be covered to reach this level of maturation with the brain-computer interface.

Neural signals collected with conventional electrochemical neural interfaces give limited information as they are ensemble readings from multiple neurons. Seamless integration by emulating the structure and properties of synapses could allow high fidelity information exchange between the brain and computer with a degree of selectivity, stability and sensitivity comparable to that of synapses. Pioneering studies on synaptic interfaces demonstrated functional synaptic connections between artificial substrates and living neurons. Janus synapses on electrodes have the potential to greatly enhance signal injection and collection efficiency by selectively interfacing with regions of neurons specialized for signal transfer. With various SAMs, it is possible to form controlled networks of single synapse-electrode junctions with different types of Janus synapses, including pre- or post-synapses as well as excitatory or inhibitory synapses. This has strong implications for the advancement of our understanding of various neural pathways as well as creating a powerful platform for next generation neural interfacing that can both stimulate and monitor the brain at each electrode channel. In addition, our understanding of the mass transport dynamics of neurochemicals within the complex space of the synaptic cleft is limited by the difficulty of gaining access inside the fragile nanospace. Direct electrochemical analysis of chemical dynamics at the Janus synaptic interface, combined with insights from fields such as nanogap electrochemistry and stochastic electroanalysis, can greatly benefit our understanding of this process.

Despite recent advances, synaptic interfaces are still in the early stages of development, and many scientific questions remain unanswered. Exploring electrode modification chemistry that ensures high stability and is suitable for long-term *in vivo* operation is crucial. The impact of surface nanotopography of SAM-immobilized electrodes on the formation and stability of Janus synapses is still an unresolved question, and expanding the library of SAMs could enhance the diversity of synaptic interfaces. Moreover, the development of devices for neuro-sensing and modulation at Janus synaptic interfaces is an essential next step in this field.

For neurosensing at electrochemical neural interfaces, devices based on MET or DET enzymatic sensors or EAB sensors can address issues of 1st generation sensors, such as the generation of harmful enzymatic byproducts. However, they have not yet reached the temporal resolution of synaptic transmission. Promising strategies to create efficient electron transfer pathways between the electrode and enzyme or aptamer include controlling the MRE orientation at the electrode surface. Such insights could be gained from the fields of biomolecular engineering and chemoselective bioconjugation.

Neuromodulation devices strive to achieve precise stimulation of neural networks. Efforts to improve the spatiotemporal resolution of these devices are ongoing. However, the implementation of iontronic chemical delivery *in vivo* requires addressing issues related to implantation. Research in this area should explore strategies for mitigating foreign body response and biofouling, as well as investigating the synergy between the Janus synaptic interface and iontronic devices.

Biological systems are able to process massive amounts of information quickly and accurately. Taking cues from neural circuits, fully aqueous circuits composed of iontronic devices powered by aqueous energy sources (*e.g.* reverse electrodialysis) have been developed with ability to mimic synaptic signals.^140,141^ Additionally, efforts are ongoing to build information processing devices using hydrogels, polymers, or macromolecules that are analogous to materials in the brain.^142^ If aqueous logic circuits can be incorporated in neural interfaces, they may be able to integrate more effectively with neurons and process information with significantly lower heat evolution than that of semiconductor circuits.

Neural interfacing is a challenging research field in which one major objective is the seamless integration of electronic circuits into biological neural circuits for bidirectional information transfer. Contributions from researchers of various sub-disciplines of chemistry are required to move closer toward this ultimate goal. In this perspective, recent developments in the seamless electrochemical neural interface have been examined to observe current trends in neural interfacing in hopes of providing inspiration to not only electrochemists but chemists from diverse fields to dive into neural interface research.

## 6. Conclusions

Electrochemical neural interface studies are shifting to afford better device performance to monitor and deliver various neurochemicals. A promising trend in this field is to employ bio-hybrid strategies in an effort to integrate foreign electrodes into the brain infrastructure. Electrochemical neural probes using synapse inducing techniques may help build neuron-electrode interfaces optimal for relaying signals to and from neurons. To achieve seamless integration of electrodes with the neural system, the physical characteristics as well as surface chemistry of neural probes have been investigated to couple with specific neuronal areas, including synapses. Surface modification strategies have also been applied to chronic detection of neurochemicals as an effort to decipher the complex relationship between neuronal signals and biological events. Finally, iontronics is an innovative approach to chemical delivery devices and is developing rapidly. Although the research explored in this perspective is still in early stages, the applicability of electrochemical methods to neural interfaces is endless. Interdisciplinary collaborative efforts to create better performing neural interfaces should continue towards establishing parallel bidirectional communication.

## Author contributions

W. C. and S. Y. outlined and wrote the manuscript. T. D. C. conceptualized and supervised the completion of the manuscript.

## Conflicts of interest

There are no conflicts to declare.

## Supplementary Material
